# Endogenous testosterone is associated with lower amygdala reactivity to angry faces and reduced aggressive behavior in healthy young women

**DOI:** 10.1038/srep38538

**Published:** 2016-12-07

**Authors:** Macià Buades-Rotger, Christin Engelke, Frederike Beyer, Brian G. Keevil, Georg Brabant, Ulrike M. Krämer

**Affiliations:** 1 Department of Neurology, University of Lübeck, Lübeck, Germany; 2 Institute of Psychology II, University of Lübeck, Lübeck, Germany; 3 Institute of Cognitive Neuroscience, University College London, London, UK; 4 Department of Clinical Biochemistry, University Hospital of South Manchester, Manchester, UK; 5 Department of Internal Medicine I, University of Lübeck, Lübeck, Germany

## Abstract

Testosterone and cortisol have been proposed to influence aggressive behavior by altering the neural processing of facial threat signals. However, this has not been investigated in direct social interactions. Here, we explored the joint impact of testosterone, cortisol, and brain reactivity to anger expressions on women’s reactive aggression in the Social Threat Aggression Paradigm (STAP). The STAP is a competitive reaction time task in which the purported opponent displays either an angry or a neutral facial expression at the beginning of each trial and delivers increasingly loud sound blasts to the participants, successfully provoking them. Strikingly, salivary testosterone at scan-time was *negatively* related to both aggression and basolateral amygdala (BLA) reactivity to angry faces, whereas cortisol had no effect. When the opponent looked angry, BLA-orbitofrontal coupling was reduced, and BLA reactivity was positively related to aggression. The latter relationship was fully mediated by bilateral superior temporal gyrus (STG) activation. Our results thus support previous neurobiological models of aggression, and extend them by demonstrating that fast amygdala responses to threat modulate STG activity in order to favor aggressive retaliation. Furthermore, our study agrees with recent evidence underscoring a fear-reducing and strategically prosocial effect of testosterone on human social behavior.

Reactive aggression is a phylogenetically ancient behavior by which organisms respond to threat or provocation with an overt intent to harm the attacker^1^. Reactive-aggressive impulses are thought to arise from subcortical structures such as the amygdala and the periaqueductal gray, which are in turn regulated by the orbitofrontal cortex (OFC) and other prefrontal regions^2^. The steroid hormones testosterone (T) and cortisol (C) have been suggested as important factors for the regulation of reactive aggression, and have been shown to act on the aforementioned brain areas by binding to androgen and glucocorticoid receptors, respectively^3^,^4^,^5^. Both hormones are considered to have a mutually opposing action^6^,^7^,^8^, and this divide is also apparent in human social behavior: T generally favors approach and aggression, while C leads to fear and avoidance^9^,^10^. This notion is supported by numerous findings in correlational^11^,^12^,^13^,^14^ and experimental settings^15^,^16^,^17^, though not all evidence agrees. For instance, some studies in women have linked high, rather than low C concentrations with aggression^18^,^19^, and high T with prosocial behavior^20^,^21^,^22^. Moreover, some controversy remains regarding whether state T and C predict aggression better than baseline values^23^. Hence, the dynamics of the relationship between these hormones and aggression are still unclear, especially in women.

Several functional magnetic resonance imaging (fMRI) studies have investigated how C and T influence the activity of brain regions involved in aggression. It has been postulated that amygdala activation in response to threat signals relates to *lower* basal C^24^,^25^, but *higher* C reactivity^25^,^26^. On the other hand, endogenous T in men has been positively associated with amygdala reactivity to threat^27^,^28^ (but see^29^), as well as with lower OFC engagement during emotion-contingent motor control^30^. In women, exogenous T seems to robustly increase amygdala reactivity to angry faces^31^,^32^,^33^, but the effects of endogenous T are less clear. For instance, van Wingen *et al*.^31^ found a positive relationship between T concentrations and amygdala reactivity to angry faces in women, while others have found none^29^,^34^. In a study using a mixed sample, endogenous T was associated with higher aggression and lower OFC activity in response to provocation^35^. Few fMRI studies have tested the joint role of both hormones in the context of aggression. Denson and colleagues^36^ found that T-to-C ratio (T:C) was linked to higher amygdala coupling with medial and lateral prefrontal regions after an interpersonal provocation in males, whereas Hermans *et al*.^32^ reported positive relationships between T:C and hypothalamus, brainstem, and amygdala reactivity to angry relative to happy faces in women. Overall, the evidence suggests that a pattern of high T and low C should lead to subcortical hyperactivity along with decreased ventral-prefrontal recruitment in the presence of threat or provocation.

Angry facial expressions are a common means to communicate hostility^37^ and are thus often used as an experimental proxy of social threat. Nevertheless, angry faces as employed in most of the commented studies rarely signal a real menace, because they are usually presented as static, context-devoid stimuli. Hence, they likely elicit briefer, weaker, or even qualitatively different neural responses than those occurring in real-world aggressive interactions. It is therefore desirable to use more realistic tasks in which angry facial expressions can be interpreted as communicative signals and neural responses to these signals can be directly related to aggressive behavior. In a previous study from our group, we developed a competitive reaction time task based on the Taylor Aggression Paradigm (TAP)^38^, which we here named Social Threat Aggression Paradigm (STAP). In the STAP, participants see a short video of their rival displaying either an angry or a neutral facial expression right before they choose the volume of an aversive sound blast to be directed at their opponent. In a sample of healthy young men, we observed that OFC reactivity to angry relative to neutral expressions was negatively related to aggressive behavior (i.e. less intense sound blasts)^39^. This finding fits studies showing that intermittent explosive disorder (IED) patients^40^ and individuals scoring high in approach motivation^41^ have reduced OFC reactivity to static angry faces. Importantly, however, we also found widespread brain activation in the last phase of angry compared to neutral trials, when no facial expression was present. This suggests that contextualized angry faces as used in the STAP frame the processing of subsequently presented information^37^ and have a longer-lasting effect than in other paradigms.

The present work had a double aim. Firstly, we investigated whether reactivity to angry faces and aggression in a direct social interaction are modulated by basal and/or acute levels of endogenous T and C. Secondly, we tested whether the findings of our previous study could replicate in a female sample. We hypothesized that morning C would be negatively related to reactive aggression, as has been shown across previous correlational studies^42^, as well as in an experimental aggression study with the TAP^43^. As to T, we predicted that it would be positively associated with aggression generally, and that T levels in the afternoon would be more strongly related to aggression than in the morning^44^. Furthermore, we expected to find a competition-induced increase in both T and C^45^,^46^. Regarding the effects of these hormones on neural structures, following the reviewed evidence we hypothesized that T would be linked to heightened amygdala reactivity to angry faces, reduced OFC activation, and a weaker coupling between these regions. We also expected to find a negative association between basal C and amygdala reactivity, whereas the pre-post task change in C should follow the opposite pattern.

We recruited 39 healthy female participants *not* taking oral contraceptives (see Methods). Subjects provided saliva samples for C and T estimation during a regular weekday, plus before and after the fMRI measurement. In addition, we obtained a pre-scan blood sample for serum T estimation. We controlled for momentary and monthly fluctuations in hormonal concentrations by having participants come during the early follicular phase of the menstrual cycle and in the afternoon (12:00–18:00), and performed the anatomical measurement on a previous day to limit scanner-related arousal. Before the functional measurement participants were introduced to their purported female opponent, a 20-year old confederate. Once in the scanner, participants played three 20-trial runs of the STAP, which was actually preprogrammed (see Methods and Fig. 1A). We probed whether participants had been provoked (i.e. if they selected louder sound blasts over time and in angry trials), and inspected for correlations between mean aggression and hormonal concentrations in the morning and at scan-time. We then tested for differences in neural reactivity to angry vs neutral faces in the decision phase (i.e. when participants decided the intensity of the punishment for their opponent) and the outcome phase (i.e. when participants were informed of whether they won or lost). Further, we examined whether aggression-related hormones and within- and between-participant variability in aggressive behavior could modulate brain reactivity to angry facial expressions. Finally, we investigated the interplay of neural and endocrine factors on aggression with mediation analyses.

## Results

### Behavioral results

**Figure 1:**
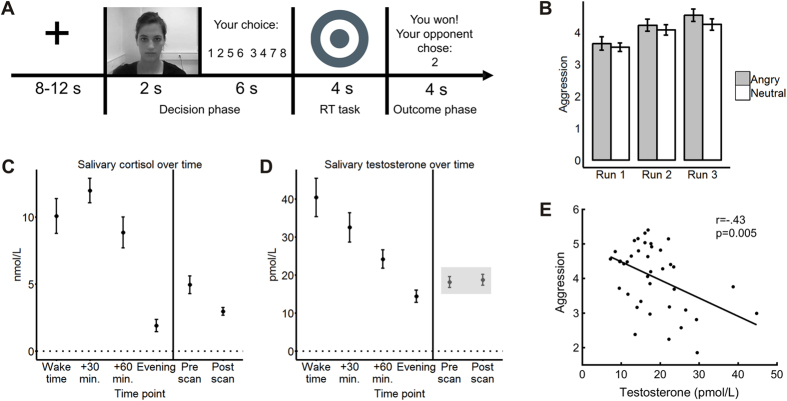
Behavioral and hormonal results. (**A**) example neutral trial of the Social Threat Aggression Paradigm (STAP). (**B**) Aggressive behavior per run and condition. (**C**) Cortisol concentrations over time. (**D**) Testosterone concentrations over time; shadowed area depicts the time points at which testosterone concentrations were related to aggression. (**E**) Scatterplot of the negative correlation between mean scan-time testosterone and mean aggressive behavior.

We observed significant main effects of time (F_2, 76_ = 21.93, p < 0.001) and condition (F_1, 38_ = 6.34, p = 0.016) on aggressive behavior, but there was no interaction between the two. As shown in Fig. 1B, aggression increased over runs and was slightly higher in angry (M = 4.13, SE = 0.16) relative to neutral trials (M = 3.94, SE = 0.14). The opponent’s punishment selection significantly affected participants’ subsequent behavior (F_1, 38_ = 20.31, p < 0.001), eliciting more aggression after high compared to low punishments (M = 4.27, SE = 0.10 vs M = 3.85, SE = 0.09). The outcome of the preceding trial (i.e. won vs lost) had an effect at trend level on aggression (F_1, 38_ = 3.16, p = 0.083), but did not interact with the opponent’s punishment selection (p = 0.340). Participants selected marginally higher punishments after losing (M = 4.13, SE = 0.10) than after winning (M = 3.99, SE = 0.15). Aggressive behavior was comparable to the male sample from our previous study (t_69_ = 1.15, p = 0.252).

For reaction times in the decision phase, there was a main effect of time (F_1.69, 64.54_ = 4.04, p = 0.030), such that participants chose the punishments increasingly quickly over runs (run 1: M = 1.33, SE = 0.09, run 2: M = 1.20, SE = 0.11, run 3: M = 1.10, SE = 0.10, all values in seconds). Pairwise comparisons revealed that only the difference between runs 1 and 3 was significant (t_38_ = 2.43, p = 0.020). The main effect of condition approached significance (F_1, 38_ = 3.55, p = 0.067), but we observed no interactions. Participants were narrowly faster to select the punishment in angry (M = 1.14, SE = 0.08 seconds) than in neutral trials (M = 1.28, SE = 0.11 seconds).

Regarding the reaction time task, there was a trend towards a main effect of time (F_1.65, 73.25_ = 3.02, p = 0.065) with reaction times being lowest in the second run, but we found no other effects.

### Hormonal results

Both ambulatory C and T levels followed a typical daily pattern (Fig. 1C,D, respectively). While T peaked at wake time and decreased steadily throughout the day, C was highest 30 minutes after awakening and decreased steeply in the afternoon. The difference between the last morning sample and the evening sample was significant for both C (t_38_ = 5.54, p < 0.001) and T (t_38_ = 4.42, p < 0.001).

Regarding state changes in hormonal concentrations, C decreased after the fMRI measurement compared to pre-scan levels (t_38_ = 4.25, p < 0.001). Only the pre-scan sample was thus taken to compute T × C interactions at scan-time, since interactive effects could be driven by the post-scan decrease in cortisol. There were no significant state changes in T, and, given that the pre- and post-scan samples were highly and significantly correlated (r = 0.77, p < 0.001), the mean of the two was obtained and used as an aggregate measure of scan-time T for correlation analyses. We also computed the pre-post percent change in T as a proxy of individual endocrine reactivity^3^. Serum and salivary T were positively correlated (r = 0.40, p = 0.011) to a degree that matched previous findings^47^. T (t_38_ = 2.11, p = 0.041) and C (t_38_ = 4.11, p < 0.001) were higher at scan-time than in the ambulatory evening sample. Morning and scan-time concentrations were moderately correlated (C: r = 0.40, p = 0.010, T: r = 0.47, p = 0.002). Mean values ± SE for both hormones at each time point are presented in Table 1.

### Hormone-behavior relationships

Aggression was not related to morning T, C or T:C, nor with the pre-post change in T (all p > 0.358). T and C did not interact in predicting aggression in the morning or before scanning (all p > 0.478). Mean T at scan-time was associated with aggression, but, against our predictions, the relationship was negative (r = −0.43, p = 0.005; Fig. 1E). We only took mean scan-time T values for further analyses with neuroimaging data.

### Neuroimaging results: task effects

**Figure 2:**
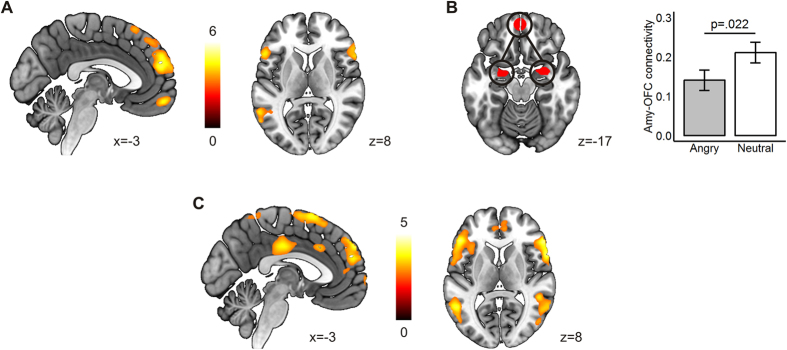
Task effects on brain activity. (**A**) Angry >Neutral effects in the decision phase (p < 0.001, pFWE <0.05 cluster-level corrected). (**B**) Amygdala-OFC connectivity in the decision phase. (**C**) Angry >Neutral effects in the outcome phase (pFDR < 0.05 voxel-level corrected, k > 10, for visualization).

The main comparison of angry vs neutral trials in the decision phase revealed clusters of activation in the medial frontal gyrus (MFG), bilateral inferior frontal gyri (IFG), the orbitofrontal cortex (OFC), the bilateral posterior cerebellum, and two separate clusters in superior (STG) and middle (MTG) left temporal gyri (Fig. 2A; Table 2a). This pattern strongly resembles that of our previous study^39^. Given that the cluster-level correction used (see Methods) can be too strict to detect meaningful activations in small structures, we separately applied a false discovery rate (FDR) corrected threshold of pFDR <0.05 at the voxel level with a minimum cluster size of k > 10. With such a threshold, we also observed activity in the left caudate (Table 2a). We defined an 8-mm radius sphere around the group OFC cluster peak to perform ROI-to-ROI connectivity analyses with the bilateral amygdala. Amygdala-OFC coupling was lower in angry than in neutral trials (Fig. 2B), but the connectivity strength was unrelated to hormones or aggression (all p > 0.491). Amygdala connectivity peaked at x = 23, y = 6, z = −18 and x = −20, y = −2, z = −25 (Montreal Neurological Institute [MNI] coordinates in mm), corresponding to the basolateral amygdala (BLA).

Again paralleling our previous study, we observed strong effects of won vs lost and angry vs neutral in the outcome phase, although there was no significant interaction between the two factors. As the won vs lost effects have been extensively investigated in previous work^48^,^49^, we report only the angry vs neutral results (Fig. 2C). In the outcome phase of angry relative to neutral trials, regardless of whether participants won or lost, there was widespread activation in IFG, MFG, and STG, as well as a cluster in the posterior cingulate cortex (PCC). At pFDR <0.05, k > 10, activation in the left amygdala was also present (Table 2b). No clusters survived multiple comparison correction in the neutral >angry contrast.

### Neuroimaging results: brain-behavior relationships

**Figure 3:**
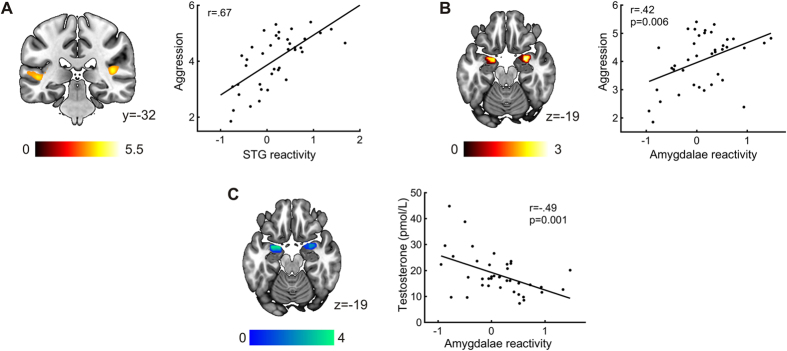
Brain-behavior and brain-hormone correlations. (**A**) Bilateral superior temporal gyrus (STG) cluster positively associated with aggression in the whole-brain contrast (Angry >Neutral) in the decision phase (left), and scatterplot of the correlation between STG reactivity and aggression (right; p-value not computed to avoid a circular analysis). (**B**) Voxels within the bilateral functional amygdala mask correlated with aggression in the Angry >Neutral contrast in the decision phase (left; p < 0.05, k > 20) and scatterplot of the correlation between amygdala reactivity and aggression (right). (**C**) Voxels within the bilateral functional amygdala mask correlated with testosterone in the Angry >Neutral contrast in the decision phase (left; p < 0.05, k > 20), and scatterplot of the correlation between amygdala reactivity and testosterone (right).

A parametric modulation analysis did not yield significant differences between angry and neutral trials, implying that modulation of neural activity by trial-to-trial variability in aggression was not different between conditions. On the other hand, whole-brain between-subject regression analysis revealed three clusters of activation positively associated to aggression in the left inferior occipital gyrus, and bilateral STG (Table 2c, Fig. 3A). That is, participants with higher reactivity to anger in these areas selected on average higher punishments. We found no negative associations between aggression and brain activity.

We also inspected for relationships between aggression and reactivity to angry expressions in the amygdala and the OFC, our a priori regions of interest (ROIs). Unlike our previous study, we found no relationships between OFC reactivity and aggression across participants, neither with the functionally defined masks nor with the cluster obtained in the decision phase (all p > 0.111). There was, however, a significant positive correlation between bilateral amygdala reactivity to angry faces and aggressive behavior (r = 0.42, p = 0.006; Fig. 3B right). This effect was maximal at coordinates x = −17, y = 0, z = −25 and x = 25, y = −2, z = −20, both in the BLA (Fig. 3B left).

### Neuroimaging results: brain-hormone relationships

A ROI analysis revealed that amygdala reactivity to anger was negatively related to scan-time T (r = −0.49, p = 0.001; Fig. 3C). We found again two clusters peaking at MNI coordinates x = −20, y = 1, z = −20 and x = 21, y = 3, z = −18, also located in the rostral BLA. These local maxima were less than 5 mm away in any direction from the aggression-related peaks, and no more than 7 mm apart from the amygdala-OFC connectivity peaks. No other area was associated with mean scan-time T in whole-brain analyses.

### Mediation results

We then selected the variables to be included in mediation analyses. Scan-time T was associated with aggression and was thus defined as predictor. As amygdala reactivity correlated with both aggression and T, we included it as mediator.

**Figure 4:**
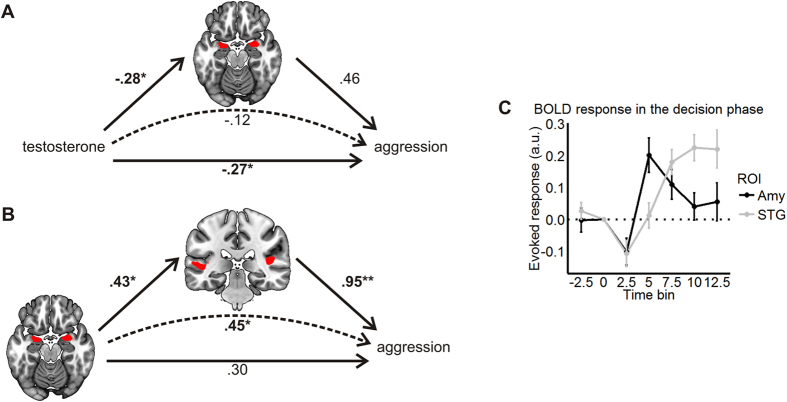
Mediational models. **(A)** Amygdala reactivity did not mediate the relationship between scan-time testosterone and aggression. (**B**) Bilateral superior temporal gyrus (STG) reactivity to angry faces mediated the effect of amygdala reactivity on aggression. Numbers are standardized regression coefficients. Solid lines: direct effects. Dashed lines: indirect effects. Numbers in bold depict paths significant at p < 0.01. The 95% BCa CI of significant paths did not include zero. *p < 0.01; **p < 0.001. (**C**) Time course of the hemodynamic response in the amygdala and the STG. Values were extracted from a 5 mm sphere limited to voxels significantly active in the decision phase. Evoked responses were adjusted for all other regressors and rescaled to 0 at onset. A.u. arbitrary units (parameter estimates).

Reactivity to angry faces in the bilateral STG was also associated with aggression. This region is, among other functions, central for action understanding in the context of threat detection^50^, and is connected to the amygdala through the inferior longitudinal fasciculus^51^. We hence reasoned that the threat signals stemming from the amygdala might modulate STG activity. To test this, we performed a mediation analysis with amygdala reactivity as main predictor and STG reactivity as a mediator. We extracted contrast values in the bilateral STG from the previous Aggression × Angry >Neutral regression analysis, and ran the model. The final mediation models are depicted in Fig. 4.

In model A, the indirect effect of T on aggression was not significant, and neither was the amygdala-aggression link. Therefore, amygdala reactivity did *not* mediate the relationship between scan-time T and aggression. In model B, bilateral STG activation fully and significantly mediated the relationship between amygdala reactivity and aggressive behavior. According to this model, amygdala reactivity led to increased STG activation, which in turn resulted in heightened reactive aggression. For this model to be realistic, the amygdala should respond faster than the STG. In order to verify this, we extracted the BOLD time course of both regions in the decision phase using *rfxplot*^52^. Indeed, the peak of amygdala activity in the decision phase came approximately 5 seconds earlier than that of the STG (Fig. 4C), strengthening the plausibility of the model.

## Discussion

In the present fMRI study we probed whether endogenous T and C influence brain reactivity to angry faces and aggressive behavior in the context of a competitive interaction in healthy young women. T was negatively associated with amygdala reactivity and aggression, but C was unrelated to neural or behavioral responses. Independently of hormonal influences, we found a positive relationship between amygdala reactivity to anger and aggression that was mediated by STG activation. Finally, amygdala-OFC connectivity was reduced in angry trials. These results show that limbic and higher-order perceptual regions interact in the processing and response to social threat, and indicate that T can reduce threat responsiveness while fostering strategic prosocial behavior in aggressive interactions.

### Aggressive behavior and hormones

We found a sizeable increase in aggressive behavior over runs, mimicking other studies with the TAP that found a highly similar pattern in provoked participants, whereas non-provoked subjects showed no change in aggression over time^43^,^53^. Moreover, participants selected moderately higher punishments in angry compared to neutral trials, as well as after high selections by the opponent, and, at trend level, after losing. Participants also became quicker in selecting the punishment over time. These results closely track those of our previous study^39^ and suggest that the paradigm successfully elicited reactive aggression.

T followed the expected decrease across the day^54^, but we did not find a competition-boosted increase in T, nor was the change in T related to aggression. Considering that these effects do not replicate well in women^3^, and, if present, occur more often after winning^18^,^55^, the fact that participants lost one third of the trials might have prevented them. Crucially and contrary to our expectations, scan-time T was negatively related to aggression. Pending replication, one might speculate that high-T participants were less intimidated by the opponent and adopted a befriending strategy over an impulsive-aggressive response. This account is supported by studies in women showing that T can reduce threat responsivity^56^,^57^ and increase prosocial behavior^20^,^58^, as well as by the lower amygdala reactivity observed in high-T participants (see “Hormonal effects on neural reactivity to anger expressions”). Importantly, T does not seem to increase pro- or antisocial behavior *per se*, but rather potentiates strategic social behavior in order to maintain or improve status and reputation^22^. Specifically, it has been suggested that T “increases the motivation to prevent a social affront”^20^. Thus, by selecting lower punishments, high-T subjects might have attempted to mitigate the opponent’s aggressiveness, whereas low-T participants might have reflexively engaged in a tit-for-tat response. However, our data does not permit to ascertain participants’ underlying motives.

C levels also followed the anticipated daily trajectory, but, unlike previous work^43^, we failed to find a relationship between basal C and aggression. Other studies have also found no such link in women^11^, and, overall, the effect seems to be more consistent in children than in older subjects^59^. The pre-post scan decrease in C levels could have been partly driven by scanner-induced drowsiness or by natural circadian variation. Complementarily, it might also be that pre-scan C was heightened due to the blood extraction procedure^60^, but probably not to scanner-related stress^61^, which had been controlled for. Unlike previous studies^18^, C did not moderate the effect of T on aggression. This might be due to our sample size, which was modest in comparison to the 100–150 participants recommended to detect T × C interactions^4^.

### Neural reactivity to anger expressions and aggressive behavior

Task effects on brain activity were very similar to our previous study, which speaks for the reliability of the STAP and suggests that the neural correlates of anger processing in an aggressive interaction are comparable in young men and women. In both cases, angry relative to neutral faces led to activation in inferior frontal, medial frontal, and middle/superior temporal gyri, regions often recruited when inferring others’ emotions and goals^62^. In the outcome phase, anger-related medial frontal activation was accompanied by larger temporal and inferior frontal clusters as well as by PCC and left amygdala activity. This reinforces the idea that angry faces in the context of an aggressive interaction provide relevant cues which influence the appraisal of later-occurring events^39^. Remarkably, we found significant anger-related OFC activation across participants, whereas, in the previous study in males, OFC reactivity was only observed in non-aggressive participants. One should in any case be cautious when directly comparing this and our previous study, as our sample size was slightly higher (7 participants more), and thus we might have reduced the likelihood of type II errors.

Basolateral amygdala (BLA) reactivity to angry faces was greater as a function of aggressive behavior. This explains the absence of amygdala effects for the angry > neutral contrast, as reactivity in this structure was heightened only among participants who responded aggressively. Although in our foregoing study in men there was no such relationship, other research has found associations between amygdala reactivity to threat/provocation and aggression in mixed-gender samples^40^,^63^. In an unpublished study from our lab, we have found that the BLA is specifically recruited when participants actively *avoid* a threatening opponent, relative to a non-threatening one. Taken together, these results suggest that the BLA signals threat relevance, flexibly facilitating either aggression or avoidance in a context-dependent manner^64^. Importantly, amygdala-OFC connectivity was reduced in the decision phase of angry trials. This decoupling was likely driven by the higher OFC reactivity to anger, possibly reflecting a regulatory or social-evaluative response to interpersonal threat^39^. Low connectivity between these two areas has been reported in IED patients relative to healthy controls^40^, and related to increased negative affect during the presentation of aversive pictures^65^. Hence, the reduction in amygdala-OFC coupling observed in angry trials might have indirectly hampered the suppression of aggressive impulses^2^.

We did not find any difference between conditions in trial-to-trial variability in aggression. This contrasts with our previous study, in which anterior cingulate cortex (ACC) activity was upregulated as a function of aggressive behavior in angry trials^39^. We did find associations however between bilateral STG reactivity to angry faces and aggression on a between-participant basis. STG activity has previously been shown to increase when focusing one’s attention on the aggressor relative to the victim of a violent conflict^66^, and when appraising unfair proposals as negative^67^. These findings, in addition to its general involvement in mentalizing^62^ and threat detection^50^, suggest that persons with higher reactivity to anger in STG might be more likely to interpret the opponent’s intentions as hostile and hence respond aggressively. Mediation analyses also support this view, as the link between amygdala reactivity and aggression was dependent on STG activation. Remarkably, the BOLD signal in the amygdala peaked around 5 seconds earlier than in the STG during the decision phase. Hence, whereas the amygdala might rapidly code for emotional salience^68^,^69^, STG activity could more slowly subserve conscious threat perception. Supporting this account, it has been recently shown that threatening faces increase both STG and amygdala activation, but only STG reactivity correlates with emotion recognition accuracy^70^.

### Hormonal effects on neural reactivity to anger expressions

BLA reactivity was negatively related to T, but did not mediate the T-aggression link. It might be that high-T individuals perceived angry faces as less threatening^29^, as suggested by studies showing that T administration mitigates fear responses^57^,^71^. Alternatively, T might generally impair emotion recognition^72^. In any case, this result indicates that high-T participants might have been better able to override defensive retaliatory responses, although the effect of T on aggression might be better explained by other factors such as testosterone-to-estradiol aromatization^73^,^74^ than by amygdala reactivity alone. It has been proposed that T buffers acute fear through its action on the BLA, which, together with the OFC, inhibits central-medial amygdala (CMA) output to the brainstem^74^. From this perspective, if high-T participants were indeed less sensitive to threat, they might have required the assumed regulatory input of the BLA to a lesser extent. Our data nonetheless suggests that the BLA does not accomplish an inhibitory or excitatory role in itself. Rather, as reasoned above, BLA activation tracks threat salience, and promotes defensive behavior adaptively.

It is worth noting that only T concentrations at scan-time were related to brain reactivity and aggression. Other studies have also found that morning and afternoon T have separable effects on the processing of threat signals, such that participants with higher morning (but not afternoon) T seem to be more attentive to angry faces^54^,^75^. Also, as mentioned in the introduction, the relationship between aggression and T appears to be stronger in the afternoon and evening than in the morning^44^, and varies across the menstrual cycle^76^. It is also unclear whether our results are entirely comparable to those of T administration studies, in which effects are likely driven by a phasic augmentation of circulating hormone levels. In the present work, scan-time T remained unchanged after the STAP and was correlated with morning T, hence probably reflecting basal endocrine function. More research is needed on how different parameters of the infradian, circadian, and ultradian hormonal fluctuation affect reactive aggression and its neural basis.

### Limitations

An important limitation of our study is that we only took ambulatory saliva samples on one regular weekday, which might not be sufficient to establish a reliable baseline^77^. Moreover, we did not measure anthropometric variables such as the body mass index, which could have also influenced our results^78^. Nevertheless, we did control for menstrual status and oral contraceptive use, and, given the prototypical temporal pattern observed in diurnal C and T levels, we deem the obtained hormonal concentrations trustable. The relatively small sample size might have anyway curtailed the sensitivity of our analyses, especially regarding C effects and TxC interactions.

It should also be taken into account that neural responses in the angry >neutral contrasts might reflect general, rather than anger-specific, emotional reactivity. Further studies could employ different facial expressions (e.g. fear) and measure participants’ subjective and/or physiological reactions in order to disentangle the valence specificity of the observed effects. It would also be meaningful to employ opponents from both genders in larger, mixed samples.

## Conclusions

We probed the influence of T and C on reactive aggression and its neural substrates in healthy young women. Our data supports the established notion that amygdala reactivity to threat is a key factor in reactive aggression, and that this effect is accompanied by reduced amygdala-OFC coupling. However, our results further suggest a complementary mechanism by which amygdala-dependent aggressive impulses would be exacerbated by activation in the STG. Scan-time T was negatively related to aggression and amygdala reactivity, suggesting that high-T participants might have felt less threatened by the opponent. All in all, our results expand existing neural models of reactive aggression, and highlight the complex nature of T-aggression relationships in humans.

## Methods

### Participants

Female participants were recruited from the university via e-mails and flyers. Exclusion criteria were present or past endocrine, psychiatric, or neurologic disorder, and use of hormonal contraceptives, which reduce circulating T levels^79^ and alter C responsivity^80^. Out of an initial sample of 43 participants, four participants were excluded from the analyses, three of them because they guessed that the paradigm was preprogrammed, and another because she was diagnosed of hyperthyroidism after the functional measurement had been performed. The final sample thus comprised 39 young female college students (mean age = 23.22, SD = 3.2), all right handed and fluent German speakers. This study was approved by the University of Lübeck ethics committee and performed according to the Declaration of Helsinki. Participants and confederate provided informed consent and received economic compensation.

### Procedure

We scheduled two appointments per participant. On the first one, only the anatomical scan was recorded. We did this to familiarize participants with the scanner environment, so that C levels in the second appointment were not influenced by situational features (scanner noise, novelty, etc.^61^). We also gave participants plastic tubes to provide saliva samples during a regular weekday (see “Saliva and blood collection”).

The functional measurement was performed on the second appointment, which was always scheduled in the afternoon (i.e. between 12 and 18) and in the first seven days after the start of the menses. We thereby aimed to mitigate circadian^47^ and monthly^81^ fluctuations in hormonal levels and in the processing of emotional facial expressions^82^. Upon arrival, we extracted an antecubital venous blood sample (see “Saliva and blood collection”). We then introduced participants to their purported opponent, a 20 year-old female confederate who arrived 5–10 minutes later than the participant. Participant and confederate read the instructions for the STAP, and they were told that the participant could see the confederate at the beginning of each trial via webcam. The confederate behaved neutrally (i.e. avoided being friendly towards the participant) and made a casual question (“Will I play with headphones or speakers?”) to make the setting more believable. The confederate was accompanied to another room to prepare for the task (she actually left) while participants provided another saliva sample. In the scanner, participants underwent a 7-minute resting-state measurement, after which the STAP started (≈30 minutes). Another 7-minute resting-state measurement was performed after the task. Once out of the scanner, participants provided an additional saliva sample and fulfilled an ad-hoc questionnaire to assess whether they had been successfully deceived. They also filled out the German versions of the Behavioral Inhibition/Approach System (BIS-BAS) scale^83^, and of the Aggression Questionnaire (AQ)^84^ to control for personality variables putatively associated with aggression and/or brain reactivity to anger^41^. Finally, participants were debriefed and compensated.

### Saliva and blood collection

Saliva was collected in plastic tubes (4 mL Cryovials from Salimetrics^®^) using the passive drool technique^85^. We requested participants to fill 4 vials on a normal weekday in which no unusual or stressful events (e.g. exams) were scheduled. Samples were collected at wake time (between 6 and 8 AM), 30 minutes later, 1 hour later, and in the evening (between 18 and 20 PM). Instructions specified that participants should fill up at least 3 mL of saliva per sample, and should refrain from: a) brushing their teeth or eating anything within 1 hour prior to collection, b) using salivary stimulants (e.g. chewing gum, lemon drops), c) consuming alcohol 12 hours prior to collection, and d) going to the dentist 48 h prior to collection^47^. Participants were instructed to store the tubes in the refrigerator after completing the saliva collection. This delays biodegradation of the saliva samples and thus reduces within-participant variability in hormonal concentrations^86^. Participants gave the tubes back on the second measurement day, which was scheduled on the same week of the ambulatory saliva collection. Two additional samples were taken before and after scanning. Saliva samples were stored at −20 °C until shipment.

Although salivary T is generally preferable as a measure of free T^47^, we extracted a blood sample (9 ml) before the functional measurement for serum T estimation. This allows to validate the salivary T values obtained, which should correlate at around r = 0.39 with serum T concentrations taken at the same time of the day^47^. The blood sample was centrifuged for 5 minutes at room temperature. Three serum aliquots of 1 ml each were extracted and stored at −80 °C until shipment. Saliva and blood samples were placed in Styrofoam boxes filled with dry ice, sealed, and shipped to author BGK’s laboratory in Manchester (UK) for analysis (see “Hormone assays”).

### Social Threat Aggression Paradigm (STAP)

We employed a modified version of the competitive reaction time task which we called the Social Threat Aggression Paradigm (STAP). In the decision phase (8 seconds), participants were shown a 2-second video of the confederate displaying either an angry or a neutral face while supposedly selecting a punishment level. After the video, participants selected the loudness of an aversive sound blast to be directed at their opponent on a 1 to 8 scale. In the reaction time task (4 seconds), participants had to press any button as fast as possible in response to a jittered target stimulus cued by an exclamation mark. In the outcome phase (4 seconds), participants were shown whether they had won or lost as well as the opponent’s punishment selection. In case they lost, they received the corresponding aversive tone. An example neutral trial is depicted in Fig. 1A. The task was programmed so that participants won approximately two thirds of the trials. Angry trials were more likely to follow trials won by the participant, and the opponent’s punishment selection was on average higher in these than in neutral trials (range 5–8 vs. 3–6, means 6.4 vs 4, respectively). Opponent’s punishment selections also increased gradually over the three runs (means: 4.2, 4.8, and 5.4). Before the task, we adjusted the loudness of the maximum punishment to each participant’s tolerance. The button distribution in the decision phase was set up as depicted in Fig. 1A to balance motor activity between both hemispheres. Participants played 60 trials (20 in each of the 3 runs), plus 4 practice trials at the beginning.

The 60 2-second videos had been pre-recorded in a separate session with the confederate. In all videos (40 neutral, 20 angry), she displaced her gaze from the keyboard to the camera keeping her expression fixed. In 10 neutral videos, the confederate kept a neutral facial expression but did not look directly into the camera. These “distracted” videos were included to make her behavior seem less artificial, and were distributed in decreasing frequency across runs (5, 3, and 2). Angry videos showed the confederate frowning and staring intensely^37^ and were distributed in increasing frequency over runs (3, 7, and 10) to make the social interaction more believable. All videos were converted to grayscale to eliminate color-related visual artifacts. All 20 angry videos and 10 normal neutral ones were rated by five female students for validation. They rated how angry, sad, concentrated and scary was the person in the video on a −9 to 9 Likert scale, and then we compared angry and neutral videos on each dimension with paired t-tests. Angry videos were perceived as angrier (t_4_ = 6.82, p = 0.002), more concentrated (t_4_ = 3.36, p = 0.028) and scarier (t_4_ = 17.94, p < 0.001), but not sadder (t_4_ = 1.05, p = 0.352) than neutral ones. Differences were in all cases normally distributed according to Shapiro-Wilk tests (all p > 0.226).

### Neuroimaging data acquisition

All scans were acquired using a 32-channel head coil mounted on a Philips Ingenia 3.0 T scanner. We acquired anatomical images with a standard T1-weighted gradient echo-planar sequence (180 sagittal slices, TR = 7.7, TE = 3.5, FOV = 240, matrix = 240 × 240 mm, flip angle = 8°, voxel size = 1 mm isotropic). Functional images were obtained with a T2*-weighted gradient echo-planar sequence for blood-oxygen level dependent (BOLD) imaging (47 axial slices per volume, TR = 2.5 s; TE = 25 ms; FOV = 200 mm, matrix = 80 × 80 mm; flip angle = 90°; voxel size = 2.5 mm isotropic). We recorded 3 consecutive runs of 200 volumes each. 5 dummy scans were performed at the beginning of each run to allow steady-state tissue magnetization.

### Behavioral data analysis

We extracted the mean punishment selection per run (1 to 3) and condition (angry and neutral) for each participant. In order to probe whether aggression increased over time, and whether participants behaved more aggressively in angry compared to neutral trials, we ran a repeated measures analysis of variance (rm-ANOVA) with factors run and condition. We proceeded identically for mean reaction times in the decision phase, and mean reaction times in the reaction time task. Additionally, we inspected whether outcome and opponent’s punishment selection in preceding trials affected subsequent aggression by means of a rm-ANOVA with factors outcome (won vs lost) and punishment (high [>4] vs low [≤4]). Sphericity corrections were applied when appropriate. Significant effects (p < 0.05) were post-hoc inspected with paired t-tests. We also performed an exploratory two-sample t-test comparing mean punishment selections in the current female sample with the male sample measured in the previous study. As in our previous study^39^, we took the mean punishment selection per participant across runs as our main aggression measure to inspect for correlations with hormones, questionnaire data, and neural activity. All analyses described in this section were performed with the *ez* package version 4.2–2 implemented in R version 3.1.3.

### Questionnaire data analysis

We first assessed the internal consistency of the BIS, BAS, and AQ with Cronbach’s alpha, and correlated scores in each of these scales with mean aggression. BAS and AQ subscales were not separately analyzed to limit the number of tests. Internal consistency was low for the BIS (α = 0.604) and the BAS (α = 0.643), but high for the AQ (α = 0.874). None of these scales were related to aggression (all p > 0.148), and thus were not used in other analyses.

### Hormone assays

For both saliva and serum samples, T and C concentrations were estimated with liquid chromatography tandem mass spectrometry (LC-MS/MS) as previously described^47^. The protocol was optimized to capture free T^87^ and C^88^ and to detect the typically low T concentrations observed in women^47^. Previously reported mean intra-assay coefficients of variation (CV) were 5.3% for T and 8.7% for C, whereas mean inter-assay CV were 9% for T and 7.8% for C^47^,^88^. The lower limits of quantification (LLOQ) were 5 pmol/L for salivary T, 0.8 nmol/L for salivary C, and 0.3 nmol/L for serum T. Serum T levels have been shown to have a mean intra-assay CV of 4.0% and mean inter-assay CV of 5.6% with this technique^47^.

### Hormone data analysis

We computed the area under the curve with respect to the ground (AUC) for salivary C and T across the three morning measurements^89^. This measure reflects total morning output of the hormones. The evening sample was compared to the last morning sample to check for the typical circadian decay in concentrations of both hormones, but was not further analyzed. We correlated mean T concentrations across the three serum aliquots with scan-time salivary values as a validity measure, but we used salivary T values for further analyses because they better reflect bioavailable concentrations of the hormone and are not distorted by individual variability in circulating albumin and sex hormone binding globulin (SHBG)^47^. We also compared C and T concentrations before and after the TAP with paired t-tests.

We inspected whether aggression was related to T, C, or T:C in the morning and at scan time with Pearson correlation coefficients, which were deemed significant at p < 0.05. Only hormonal concentrations significantly related to aggression were used for further correlation and regression analyses with neuroimaging data. Since T:C estimates might miss out on some additive effects (i.e. high-T, high-C subjects will have similar scores as low-T, low-C ones), we performed linear regression analyses to more thoroughly investigate T × C interactions^4^,^18^. We computed an interaction term by multiplying T and C concentrations in the morning and at scan time, and regressed mean aggression scores against the interaction term. In the presence of a significant interaction, we performed simple slope analyses estimating the effect of T on aggression at low (−1 SD), mean, and high (+1 SD) levels of C, as in previous work^18^. This set of analyses was performed with the *lavaan* package version 0.5–18 implemented in R version 3.1.3.

### Neuroimaging data analysis

Neuroimaging data was analyzed with Statistical Parametric Mapping 12 (SPM 12, Wellcome Department of Imaging Neuroscience, University College London, London, UK). We manually centered all volumes on the anterior-posterior commissure to ensure cross-subject alignment. Preprocessing involved slice timing correction to the middle slice, realignment to the first functional volume, coregistration of mean functional and anatomical images, segmentation of the anatomical image, normalization to the native voxel size, and smoothing with an 8 mm full width at half maximum (FWHM) Gaussian kernel. All participants showed motion parameters below 5 mm.

At the first level, we fitted event-related models with three regressors for the decision phase (angry, neutral, and distracted, 6 seconds) and four for the outcome phase (angry won, neutral won, angry lost, neutral lost, 4 seconds). Distracted and neutral trials were modelled together in the outcome phase. The reaction time task and the sound of the opponents’ punishment in the outcome phase were also modelled. We applied a 1/128 Hz high-pass filter and SPM’s built-in autocorrelation function.

At the second level, we performed a one-sample t-test contrasting angry vs neutral trials in the decision phase, and a factorial analysis in the outcome phase with the factors angry vs neutral and won vs lost, as was done in our previous study^39^. We probed whether brain activity was modulated by within-participant variability in aggressive behavior by means of a parametric modulation, as previously described^39^. We also performed a series of whole-brain regression analyses, testing whether brain reactivity to anger could be modulated by between-participant variability in aggression and endocrine function. To this end, we regressed the angry >neutral contrast against individual aggression scores, and against each aggression-related endocrine parameter. Unless otherwise noted, the statistical threshold for whole-brain analyses was p < 0.001 uncorrected at the voxel level, with a cluster-level family-wise error (FWE) correction of p < 0.05.

We also conducted analyses in our a priori ROIs, namely the amygdala and the OFC. We followed a similar procedure as in Beyer *et al*.^39^. We contrasted angry and neutral trials against baseline in the decision phase, limiting the analyses to the bilateral amygdala and left and right medial OFC (“MNI_Frontal_Med_Orb” and “MNI_Rectus”) separately. Masks were created with Wake Forest university Pickatlas^90^. These analyses were thresholded at p < 0.01 uncorrected, minimum cluster size k > 10. This way we isolated only voxels active in the decision phase. Then, we extracted mean parameter estimates for the angry >neutral contrast in the amygdala and OFC clusters obtained. We tested for relationships between contrast values at these ROIs and aggression, T, C, and T:C by means of Pearson correlation coefficients. Correlations were considered significant at p < 0.05, uncorrected. As the amygdala is a heterogeneous structure comprised of functionally diverse nuclei^74^, we more precisely localized significant effects within this brain region. To do so, we regressed behavioral/hormonal parameters on the angry >neutral contrast limiting the analysis to the functional amygdala mask at p < 0.05 (uncorrected), k > 20, and estimated the peaks’ location using the probabilistic maps included in the Anatomy toolbox^91^.

Additionally, we inspected for connectivity patterns between the amygdala and the OFC, as threat-related coupling between these two areas has been related to T^92^, T:C^36^ and aggression^40^. We performed a time-series correlation time-locked to the decision phase of angry and neutral trials using the CONN toolbox^93^. Preprocessing steps were identical as in previous work^49^. We extracted ROI-to-ROI connectivity estimates (i.e. Fisher-transformed r values) separately for angry and neutral trials and compared them with a paired t-test. We then correlated connectivity strength with aggression, T, and T:C across participants. Connectivity peaks in the amygdala were also localized with the Anatomy toolbox^91^.

### Mediation analyses

Finally, we explored whether the hypothesized links between hormones and aggression were mediated by amygdala and/or OFC reactivity to angry faces. In addition, we tested whether relationships between amygdala/OFC reactivity and aggression were mediated by changes in state hormones or by activity in other brain regions. Only variables related to aggression (our main outcome variable) and to the main predictor/s were included in the models, following established recommendations^94^. Estimation was performed with robust maximum likelihood estimation and bias-corrected accelerated (BCa) bootstrapped confidence intervals^95^. Paths were considered significant at p < 0.05 (unc.) and if the 95% BCa confidence interval did not include zero. These analyses were also performed with the *lavaan* package.

## Additional Information

**How to cite this article**: Buades-Rotger, M. *et al*. Endogenous testosterone is associated with lower amygdala reactivity to angry faces and reduced aggressive behavior in healthy young women. *Sci. Rep.* **6**, 38538; doi: 10.1038/srep38538 (2016).

**Publisher’s note:** Springer Nature remains neutral with regard to jurisdictional claims in published maps and institutional affiliations.

## Figures and Tables

**Table 1:** Hormonal concentrations.

	Mean	SE
T wake up	40.355	5.048
T + 30 min.	32.479	3.873
T + 60 min	24.145	2.425
T Evening	14.381	1.588
T pre-scan	18.122	1.419
T post-scan	18.709	1.438
Serum T	0.773	0.050
C wake up	10.072	1.294
C + 30 min.	11.961	0.920
C + 60 min.	8.837	1.160
C pre-scan	4.941	0.657
C post-scan	2.951	0.295
C evening	1.889	0.453

Values are pmol/L for testosterone and nmol/L for cortisol and serum testosterone. T: testosterone. C: cortisol. SE: standard error of the mean.

**Table 2:** Whole-brain fMRI results.

	Hem	x	y	z	k	Peak T
a) Angry >Neutral decision phase						
Inferior frontal gyrus	L	−44.5	33	0	737	6.22
Inferior temporal gyrus	R	48	8	−35	117	5.30
Cerebellum	R	23	−82	−35	152	5.26
Cerebellum	L	−22	−82	−37.5	170	5.16
Medial frontal gyrus	R/L	−4.5	55.5	20	601	5.07
Orbitofrontal cortex	R/L	−2	55.5	−15	101	4.74
Superior temporal gyrus	L	−62	−52	7.5	201	4.48
Inferior frontal gyrus	R	50.5	23	2.5	289	4.37
Middle temporal gyrus	L	−49.5	−2	−15	148	4.27
Caudate*	L	−7	8	3	24	3.66
b) Angry >Neutral outcome phase						
Inferior frontal gyrus	R	60.5	15.5	10	329	4.94
Middle temporal gyrus	R	53	−27	−5	452	4.74
Medial frontal gyrus	R/L	−9.5	63	25	262	4.65
Inferior frontal gyrus	L	−49.5	30.5	10	376	4.38
Supplementary motor area	R/L	3	10.5	67.5	344	4.38
Superior temporal gyrus	L	−57	−54.5	10	511	4.37
Posterior cingulate gyrus	R/L	5.5	−17	35	219	4.12
Amygdala*	L	−14.5	−2	−17.5	44	3.65
c) Aggression × Angry >Neutral (+)						
Lateral occipital gyrus	L	−37	−82	5	510	5.36
Superior temporal gyrus	L	−54.5	−7	2.5	216	4.61
Superior temporal gyrus	R	43	−32	10	170	4.37

Results reported at p < 0.001 (uncorrected), pFWE < 0.05 cluster-wise corrected, except *pFDR < 0.05, k > 10. Coordinates are mm in MNI space. Clusters ordered by peak T values. Voxel size = 2.5 mm isotropic. Hem: hemisphere; k: cluster size. Only each cluster’s peak is listed for clarity.
